# Topical Oxygen Therapy for Promoting Healing After Implant Placement Using Blue® M Gel: A Report of Two Cases

**DOI:** 10.7759/cureus.65258

**Published:** 2024-07-24

**Authors:** Jaweria Ansari, Girija Dodamani, Suresh Nagral, Sunil Ronad, Priyadarshani Pawar

**Affiliations:** 1 Department of Prosthodontics, Jawahar Medical Foundation’s Annasaheb Chudaman Patil Memorial Dental College, Dhule, IND

**Keywords:** peri-implantitis, chlorhexidine, oxygen therapy, healing, mucositis

## Abstract

Peri-implant diseases, such as peri-implant mucositis and peri-implantitis, are distinguished by a gradual onset of inflammation within the peri-implant mucosa, resulting in bone resorption and, ultimately, implant failure. Topical oxygen therapy is recognized for its ability to decrease inflammation, enhance blood flow, and provide a bacteriostatic effect. Utilizing oxygen-based therapy products as a local treatment for peri-implant mucositis and peri-implantitis may lead to comparable clinical results as traditional local adjuncts such as chlorhexidine, antibiotics, and antibacterial agents. This article discusses two case reports in which the Blue® M gel was utilized. In the first case, a 50-year-old female patient with a decade-long history of betel quid chewing and missing upper right first and second molars was treated with Blue® M gel to reduce the chances of peri-implantitis and promote healing following a first-stage surgical procedure for implant placement. In the second case, Blue® M gel was applied to a 56-year-old female patient who experienced pain and inflammation one week after the initial surgical procedure for implant placement to restore the missing posterior teeth on the lower right side. The use of the Blue® M gel led to accelerated healing in both instances.

## Introduction

The success and long-term viability of a dental implant are not exclusively determined by the process of osseointegration. The soft tissue enveloping the transmucosal section of a dental implant plays a crucial role in separating the peri-implant bone from the oral cavity. The term “peri-implant mucosa” refers to the soft tissue collar surrounding dental implants, as defined by Araujo and Lindhe [1]. Due to the absence of the periodontal ligament, which typically serves as a conduit for vascular anastomoses connecting the periodontium and alveolar bone, the exclusive provider of blood to the peri-implant gingiva is from the supraperiosteal blood vessels, as highlighted in the study by Berglundh et al. [2]. The levels of anti-inflammatory mediators present during the implant and periodontal surgery process are notably lower than the concentrations typically observed in traditional methods of healing. This significant variance in concentration raises intriguing questions regarding the potential impact on the overall efficacy and outcomes of implant and periodontal surgical interventions [3]. The treatment of peri-implantitis creates a wound around the implant through surgical intervention, which is anticipated to trigger an acute-phase response aimed at managing tissue damage, preventing infection, and promoting tissue repair. Prompt and efficient reactions are expected to be evident by examining peri-implant crevicular fluid, providing valuable insights into the physiological processes occurring in the peri-implant region [4].

To facilitate the process of healing while preserving the structural integrity of the healing tissue and inhibiting the colonization of microbes or infections, postoperative administration of antimicrobial agents such as chlorhexidine (CHX) mouthwash is recommended [5]. CHX has drawbacks related to its cytotoxic impact on gingival fibroblasts and its potential to disrupt tissue regeneration mechanisms [6]. The adverse effects of CHX were mitigated through the implementation of hyperbaric oxygen in the field of dentistry [7]. This type of oxygen, referred to as healing oxygen, facilitates neovascularization, the removal of harmful substances (metabolic wastes and toxins), the promotion of new blood cell generation, the enhancement of stem cell production, the acceleration of healing processes, and the elimination of bacteria [8,9]. Dr. Peter Blijdorp in the Netherlands formulated a product based on active oxygen named Blue® M gel [10]. This specific gel has been utilized to treat periodontitis, gingivitis, and mucosal ulcers [11]. The utilization of active oxygen locally to assist in wound healing and tissue regeneration appears to be a growing trend in potentially replacing CHX. The proposed concept is that the application of oxygen-containing medication enhances the healing process, subsequently diminishing postoperative discomfort. As a result, the impact of the Blue® M gel on postoperative care following implant surgery is assessed in the following two case reports.

## Case presentation

Case 1

**Figure 1:**
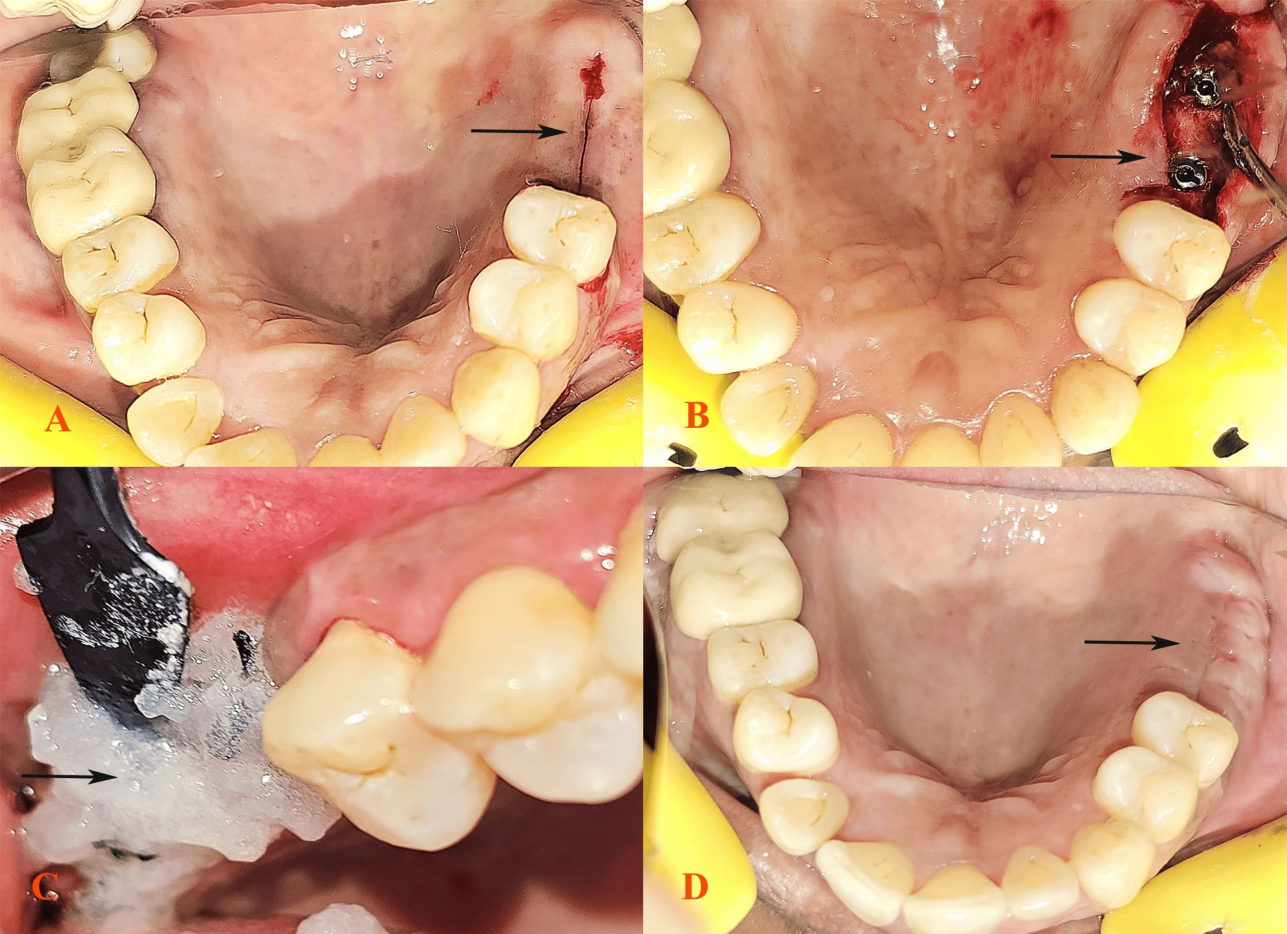
Missing 16 and 17 in the upper right quadrant (A). Implants placed successfully with primary stability (B). Application of Blue® M gel (C). Soft tissue healing at the three-week follow-up (D).

A 50-year-old female patient presented to the Department of Prosthodontics with difficulty in chewing and sought to replace her missing teeth in her upper right posterior quadrant (teeth 16 and 17) (Figure 1A). The patient had an extraction history of five years and was atraumatic. There was no relevant medical or dental history. The patient had a history of betel quid chewing for the past 10 years; approximately four to five packets per day were used to keep the quid on the right side for five minutes each time. As the patient was a frequent user of smokeless tobacco, delayed healing and postoperative peri-implantitis were expected. The patient was informed about it and motivated to stop the habit. The patient agreed and was monitored for one month. When the patient was no longer using smokeless tobacco, clinical and radiographic examinations were conducted, which confirmed normal alveolar bone health. The treatment plan included dental implant placement following comprehensive laboratory investigations to rule out hematological issues that could interfere with the surgical process. Prophylactic antibiotics were administered preoperatively. The surgical procedure was conducted under local anesthesia, during which the implants were placed successfully with primary stability (Figure 1B). Postsurgical care included detailed instructions on oral hygiene, and as the patient was an active user of smokeless tobacco for 10 years, delayed healing was anticipated. Therefore, the patient was advised to use 5-10 mL of Blue® M gel, an oxygen-enriched gel that promotes healing, two to three times a day (Figure 1C). The patient was instructed not to spit or rinse for a minimum of 30 minutes after gel application. Follow-up evaluations were conducted every week for eight weeks. At the one-week follow-up visit after the use of the Blue® M gel, significant healing was observed. Furthermore, follow-up visits revealed progressive improvement and continued satisfactory healing by the end of the third week. At the two-month follow-up visit, the implants remained stable, with excellent soft tissue healing (Figure 1D).

Case 2

**Figure 2:**
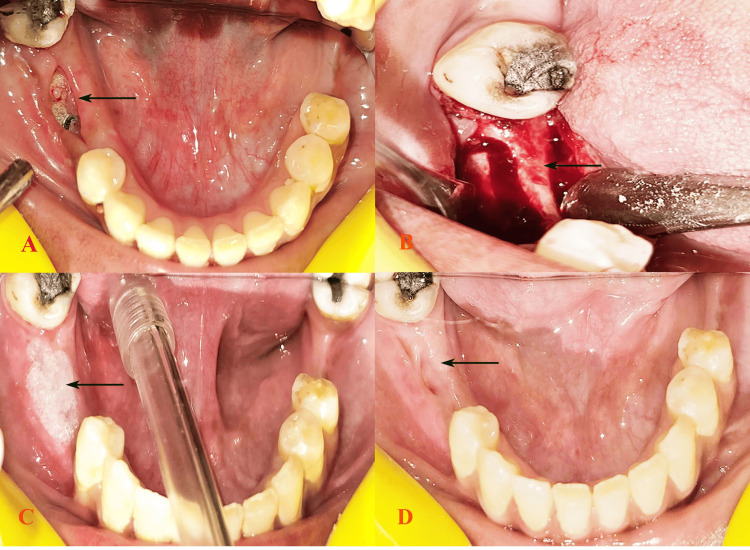
Tearing of the mucosa at the second-week follow-up (A). Clean-up of the site and resuturing (B). Application of Blue®M gel (C). Follow-up at two weeks after Blue®M gel application (D).

A 56-year-old female patient presented with bilateral missing posterior teeth in both the maxillary and mandibular jaws, indicating significant inefficiency in chewing and mastication. No relevant medical history or history of tobacco use was reported by the patient. The patient’s dental history indicated that her tooth loss was due to chronic periodontal disease. Clinical examination confirmed the absence of posterior teeth with normal alveolar mucosa, and an orthopantomogram revealed an optimal bone status suitable for implant placement. Laboratory investigations reported results within normal ranges, supporting the feasibility of implant surgery. The patient was advised to undergo implant placement, starting with the mandibular right side. First-stage implant surgery was performed following standard prophylactic measures. Postsurgical instructions included detailed oral hygiene guidelines and dietary recommendations. At the one-week follow-up visit, redness and pain were noticed at the implant site (Figure 2A), a symptomatic medication was given, and at the second week, sutures were misplaced and the mucosa was apart exposing the implant site, suggesting the development of mucositis and peri-implantitis. However, the implants were stable, and after a thorough clean-up, sutures were placed (Figure 2B). To promote healing, the use of 5-10 mL of Blue® M gel was advised two to three times a day (Figure 2C). During the next visit, one week after the use of the Blue® M gel, a significant reduction in pain and redness was observed. The patient was scheduled for weekly follow-ups for two months to monitor healing and implant integration, during which steady improvement and satisfactory outcomes were observed (Figure 2D).

Patient adherence to the care regimen, including the application of Blue® M Gel, was crucial for positive healing outcomes, suggesting its efficacy as an adjunctive therapy in dental implant procedures. This case emphasizes the potential benefits of using Blue® M Gel to enhance postsurgical recovery and implant stability.

## Discussion

After the first stage of dental implant surgery, it is crucial to take precautions to minimize the risk of early postoperative complications. These complications can include hematoma, postoperative bleeding, edema, early infection, dehiscence, and loss of primary stability of the implant [12]. Moreover, it is crucial to uphold appropriate oral hygiene practices, adhere to postoperative care guidelines, and participate in scheduled follow-up visits to guarantee the effective integration of implants and the maintenance of overall oral health.

The first case study featured the utilization of Blue® M in a female individual who had been actively consuming smokeless tobacco for a decade and who received implants for the absence of teeth #16 and #17. Though the patient was willing to leave the habit of betel quid chewing, according to Olsson et al. in a systematic review, the impact of smoking cessation before oral surgical interventions in smokers could not be ascertained [13]. Smoking increases the production of proinflammatory cytokines such as interleukin-1, leading to elevated tissue damage and loss of alveolar bone. Nicotine might impact cellular protein synthesis and hinder the ability of gingival fibroblasts to attach, thereby disrupting the healing process and increasing the risk of peri-implantitis [14]. Therefore, even after quitting betel quid chewing, the patient was instructed to use the Blue® M gel, which promoted healing and led to primary implant stability. Due to the cautious use of gel, sustained healing within two weeks was achieved, and complete healing occurred within eight weeks.

In the second case, Blue® M gel was utilized to reduce inflammation and prevent peri-implantitis. Research indicates that the typical initial healing phase lasts for three weeks, while full healing is achieved within 14-16 weeks. The Blue® M gel achieved complete healing in eight weeks. The success of implant osseointegration relies on factors such as postoperative healing, pain management, and inflammation control. Healthcare professionals administer a variety of medications, such as antibiotics, antiseptics, and anti-inflammatory agents, through local or systemic routes to facilitate the healing process.

Similar findings were reported by Tanka and Alshehri, where good healing and results were obtained in the case of a 67-year-old female patient with peri-implantitis who was treated by Blue® M gel, and results were maintained at a follow-up visit after five years [15].

The most frequent method for postoperative oral hygiene after surgery is the application of CHX, either as a mouthwash or in gel form. Research indicates that CHX can decrease the number of microbes in the area where healing occurs, but CHX does not enhance the growth of new blood vessels to support healing. Notably, CHX has harmful effects on the cells responsible for gum and bone tissue regeneration [5,16]. Neovascularization is the primary requirement to fasten the healing process.

The proliferation of capillaries, particularly those driven by the vascular component vascular endothelial growth factor is reliant on the presence of oxygen [17]. This principle underlies the application of oxygen-rich medication for the postoperative management of surgical procedures. In the context of implant surgery, the utilization of Blue® M gel, a specific pharmaceutical formulation, exemplifies this approach. Sodium perborate liberates oxygen when exposed to water and possesses antiseptic characteristics that aid in the mitigation of inflammation. In addition to its anti-inflammatory effects, the oxygen produced by sodium perborate can facilitate healing through the augmentation of cellular respiration and energy generation at the site of injury, thereby expediting tissue regeneration processes [10,11]. Lactoferrin is a versatile protein with anti-inflammatory characteristics. Through its ability to bind to iron, it hinders bacterial proliferation while also actively suppressing inflammatory reactions by engaging with immune cells. Additionally, citric acid aids in alleviating pain by maintaining an acidic environment. Furthermore, cellulose gum plays a role in forming a safeguarding barrier around the wound, leading to decreased irritation and pain through the protection of delicate tissues [10,18].

## Conclusions

Blue® M gel is a medicament abundant in oxygen that aids in the postoperative healing process, offering prompt relief from pain and inflammation. It is undeniable that Blue® M oxygen gel exhibited significant promise owing to its uncomplicated application procedure and advantageous influence on the healing of tissues adjacent to dental implants. The presented case reports supported the use of Blue® M for faster healing.
